# Computer security incident response teams: are they legally regulated? The Swiss example

**DOI:** 10.1365/s43439-022-00070-x

**Published:** 2022-11-08

**Authors:** Pauline Meyer, Sylvain Métille

**Affiliations:** 1 Faculty of law, criminal sciences and public administration, University of Lausanne, Lausanne, Switzerland; 2 UniDistance, Brig, Switzerland; 3 HDC, Lausanne, Switzerland

**Keywords:** Cybersecurity, Information security, CERT, CSIRT, Cyber incident response, Data

## Abstract

Computer Security Incident Response Teams (CSIRTs) or Computer Emergency Response Teams (CERTs) are an integral part of incident handling capabilities and are increasingly demanded by organizations such as critical infrastructures. They can hold many different skills and are of great interest to organizations in terms of cyber security and, more concretely, cyber incident management. This contribution seeks to analyze the extent to which their activity is regulated under Swiss law, considering that private CSIRTs are not regulated in the same way as governmental and national CSIRTs such as the Computer Emergency Response Team of the Swiss government and official national CERT of Switzerland (GovCERT).

## Introduction

We live in a digital society that evolves every day, and many opportunities arise from this evolution. New risks are, however, constantly discovered, and incidents continue to occur. Organizations should be prepared to prevent cyber incidents.^1^ This is what cyber security is about. There is no unique definition for this notion, but the Swiss legal definition of cyber security is the desired state in which data processing via information and communication infrastructure, in particular the exchange of data between people and organizations, works as intended (art. 3 let. a CyRV).

It is impossible to prevent all incidents from happening, despite numerous and qualitative preventive security measures.^2^ Many factors explain the prevalence of cyberattacks and incidents: The constant evolution of technology and the resulting interdependency between systems, the progressive obsolescence of devices and software, the diversity of malicious actors, the professionalization of cybercriminals and the cross-border nature of the Internet.^3^ Human beings also make mistakes while using technology. Cybercriminals can easily operate cross-border, whereas law enforcement agencies need to take additional steps and may face legal constraints.

As a result, incidents do happen and organizations must handle them, although they occur to a lesser extent in the case of an organization with appropriate preventive measures in place. Organizations should be able to count on reactive mechanisms to handle cyber incidents in the best way possible, reduce risks to a minimum and restore a secure and safe situation after their occurrence. The detection, mitigation of an incident and the recovery must be done very quickly to avoid the spread of its consequences and limit them. The benefits of calling on a skilled team like a Computer Security Incident Response Team (CSIRT) are real. A CSIRT’s main mission is to help organizations to prevent, detect, react, mitigate, recover and learn from cyber incidents. These kinds of experts in cyber incidents handling are an essential part of recognized information security standards.^4^

The overall cybersecurity level in Switzerland could and should be higher.^5^ Calling on a skilled technical team is part of the measures which could help organizations to handle cyber incidents in a more efficient way, especially for critical infrastructures. Critical infrastructures are defined in the Federal Act on Information Security (FAIS) as facilities, processes and systems essential for the functioning of the economy and the well-being of the population (art. 5 lit. c FAIS).^6^ Art. 74b of the preliminary project of the Federal Act on Information Security (PP-FAIS2), the new revised version of the law, contains a list of (critical) infrastructure subject to the obligation to report cyberattacks, which in combination with art. 5 FAIS may raise the question of the clarity of a definition of critical infrastructure, as other instruments provide different definitions^7^ and as the link between art. 5 FAIS and art. 74b PP-FAIS2 does not seem perfectly clear. Considering their role, it is particularly important that they implement the necessary measures to be protected against these cybersecurity risks.^8^

This contribution is part of a broader series of publications in the context of NRP 77 research project “Creating an ethical and legal governance framework for trustworthy cybersecurity in Switzerland”. We will present CSIRTs and their current regulation under Swiss law, and then focus on a special CSIRT, the national and governmental Computer emergency response team (CERT) of the Swiss government and official national CERT of Switzerland GovCERT.^9^ To illustrate the legal challenges they are regularly facing, we will use the example of information sharing. The present study follows a general purpose, given that there is little Swiss legal doctrine on a topic becoming increasingly important in practice.

## Computer security incident response teams

### What are they?

CSIRTs are service providers and intervention teams whose technical skills are required when cyber incidents occur, to which they will attempt to respond. They provide services in the prevention and detection of, as reaction to, cyber incidents. Their main purpose remains, in other words, to offer (mainly) technical support in case of a cyber incident (reaction), but sometimes also in terms of prevention and detection, or security quality management.^10^ The synonym Computer Emergency Response Team (CERT) is sometimes used, which today reflects more of a trademark and mainly concerns teams using the trademark; these are mainly national or governmental CERTs.^11^

A CSIRT has no unique legal or practical form. Its size also varies considerably across organizations. An organization may have an internal CSIRT, an organizational CSIRT,^12^ or a Cyber Fusion Center^13^, or even transfer some of the above-mentioned competencies to an internal Security Operations Center (SOC)^14^. Organizations can outsource some or all incident handling skills to an external (or commercial) CSIRT or to a Cyber Fusion Center, an IT service provider or vendor team/CSIRT, etc.^15^ Its constituency will significantly depend on geographic, political and contractual factors.^16^

Some of these skilled teams are public entities, in contrast to most cyber incident handling teams in Switzerland who are working as private entities. The public entities usually provide such services for authorities, such as GovCERT as a unit of the National Cyber Security Center (NCSC) who provides its services to the federal administration (and to other infrastructures), or the Military Computer Emergency Response Team (MilCERT) of the army who are both part of the federal administration.^17^ GovCERT is the national service specializing in the technical management of cyber incidents, the analysis of technical issues, the technical assessment of threats and the technical support of the national contact point, as defined by art. 12 par. 1 let. c CyRV. It is an administrative unit of the NCSC and is responsible for technical issues that come to the NCSC (art. 12 al. 1 let. c CyRV). GovCERT is a national CERT. A national CERT pursues an interest of national security. It must have some special characteristics; for example, it should be recognized as a national CERT by its government and be the point of contact at the international level. National CERTs must also carry out cyber incident management operations for computer systems of national importance,^18^ and it would be appropriate for them to have cyber risk awareness skills. They seek to safeguard interests such as the protection of national and economic security, government operations and the proper functioning of critical infrastructure.^19^ GovCERT has been performing this function since 2008 for Switzerland,^20^ together with the function of governmental CERT, responsible for remediation, recovery and rebuilding of government networks. It is the national specialized service for the technical management of cyber incidents, the analysis of technical issues, the technical threat assessment and the technical support of the Swiss Contact Point (art. 12 al. 1 let. c CyRV). GovCERT serves as the point of contact for Switzerland, participates in the coordination of the national and international incident response, and is involved in the protection of the Swiss federal administration’s network and other critical systems and networks.^21^ As we will see, GovCERT’s activity is at the moment regulated by the Ordinance on Protection against Cyber Risks in the Federal Administration (CyRV, as above-mentioned), which will be replaced by the FAIS and its ordinances. The FAIS is being amended, although not yet in force. Some provisions mentioned in the present contribution are still subject to modifications, as the legislative process is still ongoing. Art. 73a ff PP-FAIS2 provides for the competencies of the NCSC. The legal bases also refer to GovCERT’s competencies as it is the technical support of the NCSC.

### What do CSIRTs do?

The mission of CSIRTs is based on three main elements: prevention, detection and response to cyber incidents affecting their clients or their company. Their core mission being incident response, they are usually skilled in malware and forensic analysis, threat intelligence, log analysis and incident response, including development and deployment of defensive, detection and mitigation measures as well as evidence preservation, in real time when possible.^22^ CSIRTs also encourage cybersecurity awareness in terms of education and prevention in addition to their core competencies. They will encourage the prevention of future events based on past reactions.^23^ A CSIRT can also offer vulnerability handling or even security quality management services to increase the general security of an organization and indirectly reduce the number of incidents by doing so.^24^ A last important skill of CSIRTs is that they act as a single point of contact, as strong and trustworthy cooperation is necessary to handle incidents at a larger scale and to create a complete overview of threats.

### How is their activity regulated?

As mentioned, CSIRTs are often private entities which provide their services according to their clients’ needs and expectations. The function of cyber incident handler is, at least for now, not a regulated profession, regardless of whether it is an internal or an external CSIRT. The activity of a CSIRT is left to the private autonomy of the market and detailed in contracts negotiated by CSIRTs and their clients or the organization of which they are a part. They provide numerous different services, ranging from technical, strategic and legal advice to technical assistance on a client’s infrastructure and onsite.

Depending on whether they are internal to a company or whether the services of incident handling are outsourced, they will be subject to different obligations depending on the contract applicable to their activity. On the one hand, the members of an internal CSIRT within a company will be subject to the rules of the individual employment contract (art. 319 ff of the Code of Obligations [CO]). The external agreement with a CSIRT, on the other hand, may be subject to the rules of the contract of work and services (art. 363 ff CO) or the contract of the simple agency contract (art. 394 ff CO), either directly or indirectly (in the case of an innominate contract). CSIRTs will often be legally bound to their clients by complex contracts mixing frameworks with individual contracts, Services Descriptions, Service Level Agreements (SLAs) and annexes.^25^

The CSIRT is subject to some other obligations, still not specific to a CSIRT’s activity and whether the team operates solely internally or has several clients outsourcing these services. In Swiss law, the CSIRT must meet obligations related to personal data protection, such as the obligation to ensure the security of such data processed (based on art. 8 of the new Federal Act on Data Protection [nFADP])^26^. There can also be some obligations related to extracontractual responsibility (see e.g., art. 41 and 55 CO). The members of a CSIRT must moreover abide to the law as any other person, e.g., it must not commit criminal offenses, like unauthorized access to a data processing system (art. 143bis al. 1 of the Criminal Code).

Some additional regulations will apply depending on the special sector where the CSIRT operates. For example, the energy sector has adopted specific recommendations, as developed in the next section. But as we will see, critical infrastructures in this sector are submitted to minimal standards, but not CSIRTs specially.

### Special regulation: the example of the electric supply sector

The existence of private CSIRTs in general does not derive directly and expressly from the law, but is governed by private law, in the sense that contractual freedom prevails, and relations vary according to the sector.^27^ Some subsectors of the economic supply have introduced minimal ground rules for cyber incident handling within the framework of the National strategy for Switzerland’s protection against cyber risks (NCS) 2018–2022. No other critical sector has yet gone through such an examination to our knowledge, even though most of them are partly regulated, often in a fragmented and non-binding way.

Before the NCS 2018–2022 laid down the first outlines of cybersecurity regulation, several international organizations and foreign States had already started to regulate on this transversal field.^28^ The supply sector has adopted minimum standards based on these contours. The ICT minimum standard adopted by the Federal Office for National Economic Supply (FONES) provides ground rules.^29^

The standard does not expressly require the establishment of a response team, but merely states that it is necessary to clearly define the roles and responsibilities for detecting incidents (DE.DP-1) and that a response and recovery plan must be drawn up beforehand and then executed (RS.RP-1). This implies the benefits of having a team specialized for this matter. The standard requires the general procedure to be followed when detecting incidents, i.e., analysis, damage limitation, suspension of the propagation of the incident and restoration of a safe situation.

The Association of Swiss Electricity Companies (*Association des entreprises électriques suisses*, AES) has developed its own manual to adapt the Information and Communication Technology (ICT) minimum standard to the specific requirements in the electricity supply sector.^30^ The manual is voluntary and becomes binding on organizations that agree to be bound by it.^31^ The AES has provided the electricity sub-sector with a basic protection manual for operational technology. The manual is a sector-specific document that is binding for any participant who has declared it to be part of a specific contract.^32^ CERTs’ (or CSIRTs’) competencies are based on three main activities, namely that it provides proactive services, reactive services, and security quality management services according to this document, which partially considers several international recommendations.^33^

After the minimal standard of the FONES and the manual of the AES, some additional changes have recently been undertaken. The Federal Act on Electric Supply is being modified in the frame of the revision of the FAIS, as the Swiss Federal Office of Energy concluded that the electric supply sector missed a binding regulation.^34^ A new art. 8a of the Federal Act on Electric Supply is consequently being adopted. This provision states that grid operators, generators and storage agents must take steps to adequately protect their facilities against cyber risks. It also states that the Federal Council can extend this obligation to other important parties. This general obligation includes preventive and reactive measures, which should be detailed in an ordinance.^35^

Not all other critical sectors have regulated with such a far-reaching legally binding act for ensuring cybersecurity. The modification of the Federal Act on Electric Supply is also very general, and we can ask ourselves if it is sufficient for binding minimal standards. We will have to wait and see how the ordinance details the new general obligation, for example by the preparation of an incident handling plan or by the clarification of the roles. Creating an obligation to count on a competent cyber incident handling team would in our opinion be welcomed, as these teams provide real added value for organizations. Time will tell if they are necessary for organizations to meet their cyber security obligations.

The other sectors are more or less regulated. For example, the Finance sector is heavily regulated by the Swiss Financial Market Supervisory Authority (FINMA). The telecommunication subsector is regulated by the Telecommunications Act (TCA) which requires telecommunications service providers to combat any unauthorized manipulation of telecommunications equipment by means of ICT transmissions (art. 48a) and gives the Federal Counsil, who delegated the task of drawing up technical and administrative security regulations to the Federal Office of Communications (OFCOM), the responsibility for issuing provisions on the security of information and telecommunications infrastructures and services. The health sector is regulated in a fragmented way, as some questions must be regulated on a cantonal level and others on the federal level.

### The example of information sharing

As this contribution does not aim to detail the whole legal framework, it will limit itself to illustrating how legal challenges affect CSIRTs’ practice with the example of information sharing, one of their daily and main activities. To complete their mission, CSIRTs engage in cooperation with many actors.

For a rare but important example, a bank’s internal CSIRT receives an alert indicating an unauthorized access into a protected system. After confirming that an intruder has successfully penetrated the system, the CSIRT understands that the malicious actor has released malware into the network to encrypt and steal data.

In such a case, the CSIRT shares information internally (or with its client when the services are outsourced). The team alerts the people in charge of decision-making within the organization. It may alert users of the impacted activity when an incident is discovered. The CSIRT also cooperates with different internal stakeholders during the management of an incident (for example with the legal department as personal data is involved). Finally, it provides information to its supervisor at the closure of the process of incident handling.^36^

The CSIRT can share information externally both nationally and internationally and can face both optional (with communities of CSIRTs for their interests, or with telecommunication providers) and mandatory (e.g., by being responsible for filing complaints, reporting to a supervisory authority or reporting certain cyber incidents) announcements^37^. The CSIRT can be the one making at least some reports for the organization that the legal team, or the communication team of the organization are more likely to address externally. In our example, as the infrastructure victim of the attack is a bank, the CSIRT is likely, depending on the structure of the organization, to proceed to elaborating a report for legal teams to proceed to the mandatory reporting to the FINMA (if the conditions of art. 29 al. 2 FINMASA are met).^38^ The organization also reports the data security breach to the Federal Data Protection and Information Commissioner (FDPIC) as the breach is likely to result in a high risk to the personality of the data subjects, and to the data subjects if they can take measures, based on art. 24 nFADP.^39^ Furthermore, once the PP-FAIS2 is enacted, the bank will also have to report the cyberattack to the NCSC (art. 74a ff PP-FAIS2, specially art. 74b lit. e and 74 d al. 1 lit. c PP-FAIS2). It is possible, but not mandatory, to assign these competencies to the CSIRT or, at least, to assign to the CSIRT the competence of elaborating the report for the legal team, which is more likely to handle the mandatory report to the NCSC.^40^

Considering the above-mentioned announcements, the report to the NCSC could be one of the CSIRT’s functions, being the most qualified within the organization concerning cyberattacks. The team is otherwise at least working with the legal team competent for the reporting obligations. Critical infrastructure’s operators must report cyberattacks that put the infrastructure in danger, if a State-actor is involved, if it could lead to data leak or manipulation, if it has spent more than 30 days undiscovered or if the malicious actors threaten the operator (art. 74d PP-FAIS2). In our case of the bank victim of a cyberattack, data has leaked. In consequence, this must be reported by the organization. By reporting the attack, the critical infrastructure’s operator shares data with the NCSC about the critical infrastructure, the kind of cyberattack, its development, its consequences and the measures considered to remediate to it (art. 74e al. 1 PP-FAIS2).^41^ The NCSC would probably give some recommendation to the bank to help remediate the incident and recover, so we can see that the information flow is bidirectional.

CSIRTs are also likely to receive information. They may not always have, for instance, the answers and the knowledge necessary to patch every vulnerability discovered, or to remediate alone to every cyberattack and -incident that is reported to them. Most CSIRTs are part of specialized communities whose members share information to help one another, as it is possible that a CSIRT, e.g., in another country, has faced the same issues as a CSIRT in Switzerland. Another CSIRT, in Switzerland or somewhere else, might, furthermore, have some valuable information about measures to handle a certain type of attack or to respond to a special malware. The other CSIRT could share strategic or operational information with the CSIRT working in Switzerland. This is the reason behind CSIRTs’ communities and if the CSIRT in our example is a member of such a community, it can benefit from information coming from trusted partners.^42^ A CSIRT is also likely to receive information from its client(s), which is necessary for the team to handle an incident or a vulnerability. When a CSIRT serves a community of clients, some information coming from one client could be useful to others.

Information sharing raises the question of sharing protected data among others. A CSIRT is likely to share technical, secret, personal data. It can typically share technical data like indicators of compromises, secrecy covered data (often related to manufacturing or trade secrecy, art. 362 of the Criminal Code) or personal data linked to addressing resources, IP addresses or domain names.

Sharing information may expose CSIRTs to liabilities in certain cases, which is why it must carefully respect all kinds of regulations.^43^ The various secrets are likely to constitute obstacles to information sharing. When a CSIRT needs to have access to addressing resources,^44^ which constitute personal data, it must respect the data protection regulations, usually the Federal Act on Data Protection (FADP), which will soon be replaced by the nFADP. It means that the CSIRT will have to be careful to respect the different applicable provisions to avoid unauthorized communications.

It is easier to share data with authorities as this information flow must be more regulated by law. Information sharing with other stakeholders could, however, lead to questions related to the type of information shared. For example, some data could be covered by secrecy. In our example, the bank secrecy (art. 47 al. 1 lit. c of the Federal Act on Banks, LB) or the commercial secrecy (art. 162 of the Swiss Criminal Code) could limit the information sharing.

**Fig. 1:**
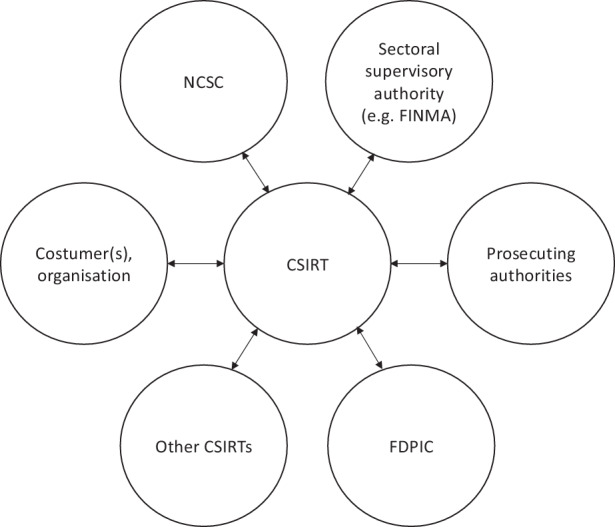
Possible information flow around CSIRTs

This communication flow coming from and to the CSIRT (Fig. 1) can be seen as a challenge as the team has to know which data it can share with whom and under which conditions to make sure it does not break the law with an unauthorized communication^45^. Some tools are used in practice to avoid such unauthorized communication, like the classification scheme Traffic Light Protocol (TLP), which allows the exchange of information to be accompanied by requirements specifying to whom the information must, may or may not be transmitted (e.g., whether information can be freely accessible, whether it can only be transmitted to a specific sector and so on), or non-disclosure agreements (NDAs).^46^

## The specific case of govCERT

### A particular CSIRT

The activity of GovCERT is subject to stricter regulation compared to other CSIRTs. GovCERT is a federal body that must base its activity on a legal basis under the principle of legality (art. 5 al. 1 Cst.), as does the NCSC. The first sub-principle is the rule of law, namely that the State may not act in violation of the law but must act in accordance with the purposes, means, conditions and circumstances authorized by the law. The second sub-principle is the reservation of the law, according to which the state may not act if the law does not provide for any action.^47^

The main goal of the governmental CERT is to protect national security, the proper functioning of government operations, the ability of critical infrastructure to perform their functions and to participate in the cyber hygiene in the Swiss address space. Its incident response competencies will target the federal administration’s and critical infrastructure’s IT networks and systems. It has broader awareness-raising competencies and is the point of contact for national and international relations.^48^

Clear legal bases are lacking today for GovCERT’s mission. The FAIS is necessary, above all in its revised version, as GovCERT needs new competencies authorized by the law to be able to handle the future obligation for critical infrastructures to report cyber-attacks.

GovCERT’s competencies are more limited when it comes to the general population, as art. 73a PP-FAIS2 **implicitly** states that it:

- Participates in raising public awareness of cyber risks.
- Warns against cyber risks and the vulnerability of IT resources.
- Participates in the publication of information on cybersecurity and on preventive and reactive measures.
- Carries out technical analyses aimed at assessing and eliminating cyber risks
- Deals technically with reports of cyber incidents and vulnerabilities that can be reported by anyone to the National “Contact Point”. GovCERT analyzes the reported incidents and vulnerabilities. The NCSC will issue a recommendation considering the analysis and feedback from GovCERT if the reporter wishes (art. 73b al. 2 PP-FAIS2). The law states that this recommendation is limited to cases which require no further analysis or clarification. That means that if a victim organization reports a new interesting *modus operandi* or reports new Indicators of compromise (IOCs) and that GovCERT doesn’t have enough information to issue a recommendation, the service gathers and shares the information to increase protection (e.g., by sending new Phishing URLs to browser providers and anti-phishing working groups), and analyzes the new *modus operandi* or the IOC for Threat Intelligence. That also means that GovCERT doesn’t compete with the private service providers for incident response and vulnerability handling. The other competence of GovCERT in these cases seems to be the provision to authorities and organizations with information on cyber incidents and vulnerabilities to combat cyber risks (art. 73b al. 3 PP-FAIS2).

GovCERT works more intensively with the Swiss network of critical infrastructure operators, including the members of the closed customer group of the former Reporting and Analysis Centre for Information Assurance MELANI^49^ and supports these operators of critical infrastructures, on which the population depends and whose security and resilience must be particularly maintained (art. 74 al. 1 PP-FAIS2).^50^ It provides a secure communication system, technical information on cyber risks and vulnerabilities, recommendations on preventive measures and technical tools and instructions aiming to detect cyber incidents (art. 74 al. 2 PP-FAIS2). GovCERT can analyze threats that it finds by itself or that are reported from other organizations and provide additional insights and detection measures. Such information is also called Threat Intelligence and consists of TTPs (Techniques, Tactics and Procedures) and IOCs. GovCERT also advises and assists critical infrastructure’s operators in cyber incident handling and remediation of vulnerabilities. It helps private operators only if no equivalent support on the market can be found in the right time (art. 74 al. 3 PP-FAIS2). GovCERT has additional competencies when it comes to the obligation to report cyberattacks for critical infrastructures.^51^ GovCERT helps the NCSC to identify *modus operandi* at an early stage, alert potential victims and recommend them prevention and defense measures in this frame (art. 74b PP-FAIS2).

GovCERT’s team can take some other special measures. GovCERT can request the OFCOM or SWITCH the technical and administrative blocking of a domain name from the registry for 30 days if there are suspicions of misuse of a domain name, as it should be recognized as a service fighting cybercrime. The domain name will then be blocked, which means that the domain name cannot be attributed and any traffic to that domain will not be routed to all sites attached to it that endanger the network.^52^ GovCERT can also ask the registry to “suspend or modify a domain name technically by deleting the related name servers in the zone file, replacing them with new name servers or not re-entering them after their deletion”, to correct or erase WHOIS information, to redirect, for analysis, flow transiting at a domain name and so on (art. 30 al. 3 of the Ordinance on Internet Domains, OID).^53^

### The example of information sharing

#### Introduction

GovCERT has wide cooperation and information exchange competencies because it is the national CERT. Many different information flows exist. GovCERT’s activity is subject to the principle of legality as above-mentioned, so the law must provide for the exchange of information, which can consist in protected data such as personal data or data submitted to secrecy. The FAIS, the provisions of which are currently being amended, serves as the legal basis for these different information flows. Provisions in this law also serve as a legal basis for the communication of personal data as required by art. 36 nFADP.^54^ The personal data communicated in this context can typically consist of personal data linked to addressing resources as mentioned above. These resources may relate to criminal or administrative proceedings and may therefore constitute sensitive data, the processing of which must be provided for by a legal basis in the formal sense (art. 34 al. 2 nFADP), which is why a formal legal basis should enable GovCERT to process and communicate them.

#### Cyberattack reported

For example, the NCSC and GovCERT are informed by a critical infrastructure’s operator that a software vulnerability has been exploited by malicious actors. The software might be used by many other organizations in Switzerland. GovCERT receives information through the report based on art. 74a ss PP-FAIS2. In consequence, GovCERT exchanges information with the operator reporting to identify *modus operandi*, potential victims and to support the critical infrastructure during the incident response (art. 74a PP-FAIS2). When it is useful for the protection of critical infrastructure against cyber risks, GovCERT also shares data with the rest of its constituency or with other members of the community it serves (art. 76 PP-FAIS2). This communication is useful in our example, as other operators of critical infrastructure are likely to also use the vulnerable software and may be at risk of having the same intrusion.

If GovCERT needs to know about IP addresses for its activity, it can also communicate with telecommunication service providers. Personal data shared in this context may consist in addressing resources and other personal data linked to them. They can be shared only if it is useful for the protection of critical infrastructure against cyber risks (art. 76 al. 3 PP-FAIS2).^55^ In the process of identifying and notifying a victim, GovCERT usually does not receive information directly from service providers. The service uses Open-Source Intelligence (OSINT) to gather information (e.g., by a certificate on an IP address revealing the name of the organization) or collaborates with the police who can do the lookup and then ask GovCERT to inform the victim. Otherwise, it is the Internet service provider (ISP) who contacts the customer and asks them to contact GovCERT.

If the specific case requires it, GovCERT can share the necessary information with the federal Intelligence Service. It shares information regarding a reported incident when it can be helpful to detect in time and prevent threats to internal or external security, to assess the level of threat or to provide an early warning service for the protection of critical infrastructure to the intelligence field in accordance with art. 6 al. 1 let. A, 2 and 5 of the Federal Act on the Intelligence Service (IntelSA) (art. 73c al. 1 PP-FAIS2).^56^ NCSC also shares information with the Intelligence Service regarding the number of cyberattacks, their type and scope, as well as its technical analysis of cyber risks to help the Intelligence Service in its competencies based on art. 6 al. 1 lit. a, 2 and 5 IntelSA (art. 76a al. 1 PP-FAIS2). The NCSC’s unit also gives to the Intelligence Service access to information on offenders’ identity and *modus operandi* as the NCSC has an important source of information about cyber incidents and threats (art. 76a al. 2 PP-FAIS2). This remote access contains technical information as well as personal data such as addressing resources (e.g., domain name, IP addresses or e‑mail addresses used abusively) or information on financial transactions (bank accounts, IBAN numbers, etc.).^57^

GovCERT, with the NCSC’s approval, can choose whethre it reports or not to prosecuting authorities when it comes to the discovery of a potential offense during an incident’s analysis (art. 73c PP-FAIS2). Its decision is based on the gravity of the potential offense and on the balancing of the interest of the State in criminal prosecution against the interest of the person making the alert in the confidentiality of the information.^58^ GovCERT will probably not report for offenses like unauthorized access to a data processing system (art. 143bis of the Criminal Code). It is likely that more serious offenses such as extortion (art. 156 of the Criminal Code) will be reported. The question will be more relevant for offenses with a severity level between these kinds of offenses, like damage to data (art. 144bis of the Criminal Code). However, the information communicated cannot be used against the person reporting (as states art. 73c al. 3 PP-FAIS2 as a concretization of the principle *nemo tenetur*). The prosecuting authorities also have access to information on offenders’ identity and *modus operandi* (art. 76a al. 3 PP-FAIS2).^59^ The PP-FAIS2 seems to be lacking some clarity regarding communications to the Intelligence Service and prosecutors, which could lead to more challenges in GovCERT’s practice if it is not amended during the legislative process. In particular, GovCERT may find itself in a confrontation between an obligation to communicate information to the Intelligence Service in the circumstances of art. 6 al. 1, lit. a, al. 2 and 5 of the IntelSA, which can be described as lacking clarity, and a wish not to breach a non-disclosure contracts with the interlocutors who gave him such information.

#### Vulnerability reported

As another example, a trusted partner of GovCERT on the international level tells the team about a vulnerability (not known as having been exploited) that is present in an IT product used by many organizations in Switzerland. The partner has figured out how to patch it and shares recommendations with GovCERT or a specialized team within NCSC (Vulnerability Management Team). The team can then share technical information, tools, instructions and recommendations with critical infrastructure’s operators (art. 74 al. 2 PP-FAIS2).^60^ If GovCERT knows about such a vulnerability, it can share data also at the international level with foreign or international services specialized in cybersecurity when it is necessary for the accomplishment of tasks comparable to those of the NCSC (art. 77 al. 1 PP-FAIS2). If personal data are shared, the conditions of art. 16 f. nFADP must be respected, which can differ depending on the circumstances of each communication. GovCERT must be careful to use the correct guarantee based on these provisions.^61^ When information is necessary for a foreign procedure, art. 77 PP-FAIS2 cannot be used to bypass the provision on mutual assistance (art. 77 al. 3 PP-FAIS2).^62^

In this case, the NCSC can interact with manufacturers, interested entities, the public and potential victims. It immediately informs manufacturers when it discovers a vulnerability so they can patch it. If they do not, the NCSC can publish the vulnerability and information about the product(s) affected so the users can take the appropriate measures to prevent cyberattacks (art. 73b al. 3 PP-FAIS2).^63^

The public also gets other information concerning cybersecurity and different measures (art. 73a lit. a, b and c PP-FAIS2). GovCERT can, with the NCSC, publish or share data regarding reported incidents or vulnerabilities with authorities or interested organizations when it can help prevent or fight cyberattacks (art. 73b a. 2 PP-FAIS2). This information can contain personal data only when it concerns misused identifiers and addressing resources (for example in cases of misuse of a logo in a phishing attack) and the data subject has given his or her consent (art. 73b al. 2 *infine* PP-FAIS2).^64^ The NCSC alerts potential victims when a cyberattack is reported and recommends the appropriate prevention and defensive measures (art. 74a PP-FAIS2). It also informs the person(s) affected by the founded suspicion of abusive use of addressing resource(s) or identity theft so he or she can take the necessary measures or file a criminal complaint (art. 75 al. 3 PP-FAIS2)^65^.

#### Conclusion

**Fig. 2:**
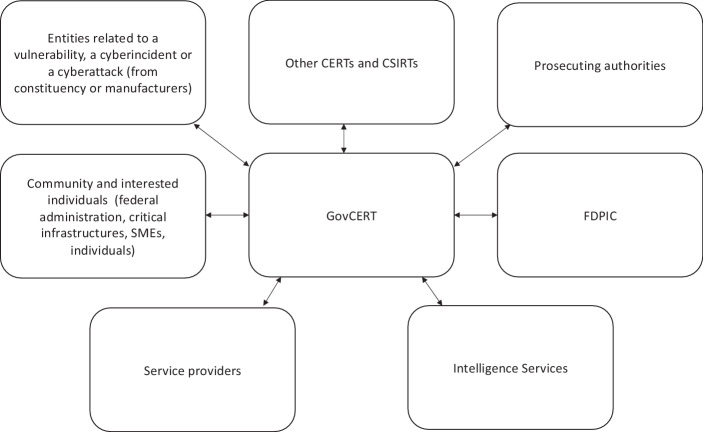
Possible information flow going through GovCERT

GovCERT is at the center of much flow of information, even if there are naturally also information flows without its involvement (Fig. 2). The federal unit can receive or share data, even if it is considered as personal data (art. 16 f, 36 nFADP and the applicable provisions of the PP-FAIS2) or data covered by secrecy (art. 73c al. 4 FAIS2 and art. 320 of the Swiss Criminal Code). It must, however, be careful and respect the provisions like the FAIS as above-mentioned. GovCERT must respect the nFADP when it is sharing personal data. It can typically only share personal data if there is a legal basis for doing so. In the same way of thinking, the unit can only carry out transborder communications of personal data based on one of the guarantees of art. 16 f. nFADP. If GovCERT wants to share data covered by official secrecy, it can only proceed so if its superior authority agrees (art. 320 al. 2 of the Criminal Code). All these provisions need to be respected to preserve the different stakeholders’ and subjects’ interests.

The team should be careful when it comes to information sharing, as it should respect the above-mentioned data and secrecy protection regulations. GovCERT should be careful when it shares information with the Intelligence Service or the prosecution authorities as the law is not yet clear enough on the circumstances of these information flows. The FAIS in its revised version is not clear whether the NCSC can share secrecy-covered information with the Intelligence Service, or the prosecution authorities based on art. 73c PP-FAIS2 in every situation (when there is a cyber incident or also when it comes to information about cyberattacks). It seems that art. 73c PP-FAIS2 provides GovCERT with the possibility to share personal data regarding cyber incidents, vulnerabilities and cyberattacks against critical infrastructures to the Intelligence Service and the prosecution authorities, but it lacks coherence.^66^ Problems like these should be addressed during the legislative process.

GovCERT must also be careful when it comes to information exchange with partners, typically on an international level, as not every partner can be trusted equally. The team should typically consider the reliability of received information and security issues.

## Conclusion

The legal framework surrounding GovCERT’s activity is, on the one hand, beginning to take shape, although in practice it remains unclear. Its presence seems essential for the proper management of incidents of national importance. We welcome the evolution in the PP-FAIS2 regarding the clarification of the role, skills and organization of GovCERT and the NCSC. The project will still have to be amended to be clear enough, but adaptations are being implemented to legitimize GovCERT in cyber incident handling and information sharing.

Private CSIRTs, on the other hand, have few regulations defining their mission, since contractual freedom and party autonomy prevail. They remain subject to some general regulations, like privacy regulations, that have a great influence on the work of private CSIRTs. This raises the question, especially in critical sectors, whether a CSIRT should be explicitly mandatory by law considering the high risks linked with targeting critical infrastructure and the new obligations for critical infrastructure’s operators like the obligation to report cyberattacks. Even if NDAs, classifications and special agreements are being put in place in the CSIRTs’ community to share information securely,^67^ it might be interesting to regulate their position to optimize the information sharing between all the stakeholders and to increase the incident handling capabilities of critical infrastructures.

If we take a step back from CSIRTs’ activity, critical infrastructures, composing a community with especially high needs in terms of cybersecurity, deserve special protection against cyber incidents, as they are nowadays heavily dependent on ICT, and the whole population and society depends on them. The current level of cybersecurity in Switzerland seems not to be satisfactory. A regulation has begun in the electric supply sector in the sense that requirements are being established for cyber incident management, but it remains very general. The rest of the regulation in this sector is not necessarily binding. CSIRTs or, at least, people specialized in cyber incident handling are not legally necessary. There are no general minimal standards in terms of cybersecurity and therefore operators in different sectors do not ensure a harmonized minimum level of cybersecurity.

A few solutions could be imagined. Every critical sector could be subject to binding norms that require processes and clear roles in preventive and reactive measures assuring cyber incident management, on which basis more concrete obligations should be figured out. As a solution, we could imagine that art. 6 ff FAIS could apply to all the operators of critical infrastructure as a general minimal cybersecurity regulation, based on which the different critical sectors could then create their own specific standards to take into consideration their sectorial specificities. This solution, risky in the sense that the law cannot evolve as fast as the technology, could unfortunately lead to a “check the box” regulation by critical infrastructure’s operators. Incentives should also be well thought before implementing such binding standards, in order to respect the autonomy of the different sectors.

These basic requirements could be considered, limited or not to the legal requirement for membership in a CSIRT, and could for example apply to all critical infrastructure’s operators. To avoid the above-mentioned problems with a binding minimal standard, other legal incentives could be imagined, like the obligation for critical infrastructures to accept a penetration test or an audit by order of the NCSC, or to obtain a certification which must be regularly renewed. The legislator would thus respect the autonomy of the critical sectors in elaborating minimal standards. This solution also allows critical operators to freely choose their cybersecurity measures and adapt them considering the technological evolution by avoiding a “check the box” regulation.

## Footnotes

1 Cyber incidents are currently defined at art. 5 lit. d PP-FAIS2 as events occurring during the operation of IT resources which may result in a breach of confidentiality, integrity, and availability of information or in the traceability of its processing. Cyberattacks are part of cyber incidents, with the precision that they hold a malicious intent (art. 5 lit. e PP-FAIS2). The Federal Act on Information Security (FAIS, *Loi sur la sécurité de l’information, Informationssicherheitsgesetz*) is supposed to come into force in 2023. It is currently being revised to introduce the obligation to report cyberattacks on critical infrastructure. When the authors refer to “FAIS”, they mean the initial version of the law entering into force in 2023, whereas when they refer to “PP-FAIS2”, they mean the preliminary project of the revised version of the law that is expected to pass through Parliament at the end of 2022.

2 Swiss Federal Counsil (2017) [21], Message du 22 février 2017 concernant la loi sur la sécurité de l’information, FF 2017 2831.

3 Dsouza Z (2018) [4] *Are Cyber Security Incident Response Teams (CSIRTs) Redundant or Can They Be Relevant to International Cyber Security?*, Federal Communications Law Journal, vol. 69, p. 204 s.

4 See for example ISO 2700x, NIST Cybersecurity Framework, COBIT, BSI Standard or ENISA Guidelines; Swiss Federal Counsil (2019) [24] Rapport “Sécurité des produits et gestion des risques de la chaîne d’approvisionnement dans les domaines de la cybersécurité et de la cyberdéfense” en réponse aux postulats Dobler 19.3135 “Acquisitions de l’armée. Avons-nous la maîtrise de la cybersécurité?” et 19.3136 “Infrastructures critiques. Avons-nous la maîtrise des composants matériels et logiciels?” du 18 mars 2019, p. 10 s.

5 For an example of a recent study showing a similar opinion, Ethos (2022) [9] *Étude Ethos—Responsabilité numérique des entreprises du SMI Expanded*, p. 3.

6 For the definition of the law, cf. footnote 1.

7 See e.g., art. 6 al. 1 lit. a n. 4 of the Federal Act on the Intelligence Service (IntelSA) or the definition in the Strategy for the protection of critical infrastructures, Swiss Federal Counsil (2017) [23], Stratégie nationale pour la protection des infrastructures critiques 2018–2022 du 8 décembre 2017, FF 2018 499.

8 Swiss Federal Counsil (2019) [25], Plan de mise en œuvre de la stratégie nationale de protection de la Suisse contre les cyberrisques (NCS) 2018–2022, May 2019, p. 5.

9 For the notions of national and governmental CERT, cf. *infra *2.1.

10 ENISA (2020) [8] How to set up CSIRT and SOC, p. 6; ENISA (2010) [6] Incident Management Guide, p. 12; OFEN (2021) [20] Cyber-Sicherheit und Cyber Resilienz für die Schweizer Stromversorgung, p. 133; Bada M et al. (2014) [2] *Computer Security Incident Response Teams (CSIRTs)—An Overview*, p. 6 s. Security quality management services “augment existing and well-established services that are independent of incident handling and traditionally performed by other areas of an organization, such as the IT, audit, or training departments […] the CSIRT’s point of view and expertise can provide insight to help improve the overall security of the organization and identify risks, threats, and system weaknesses. These services are generally proactive but contribute indirectly to reducing the number of incidents”, West-Brown et al. (2003) [26] Handbook for Computer Security Incident Response Teams (CSIRTs), p. 24 s.

11 ENISA (2010) [6] Incident Management Guide, p. 12; Bada M et al. (2014) [2, p. 6]; Johansen G (2020) [15] Digital Forensics and Incident Response, Incident response techniques and procedures to respond to modern cyber threats, 2nd edition, p. 13. The trademark is owned by the CERT/CC of the Software Engineering Institute of Carnegie Mellon University, which is competent to coordinate vulnerability and incident management actions and shares best practices, but CSIRTs outside the United States of America should consult their own trademark council if they wish to enter into a licensing agreement, https://www.sei.cmu.edu/our-work/cybersecurity-center-development/authorized-users/ (consulted on the 2nd of November 2022).

12 An organizational or internal CSIRT handle incidents on internal networks of an organization, Dsouza (2018) [4, p. 211].

13 This kind of center usually regroup CSIRT, SOC and threat intelligence specialists to fusion their respective competencies, cf. Johansen (2020) [15, p. 38].

14 A SOC is the focal point for security operations and computer network defense for an organization in terms of monitoring and incident handling. It is a center for all security-relevant services in the IT environment of a company and integrates, monitors and analyzes all security-relevant systems as company networks, servers or internet services. Usually, the SOC is mainly concerned with operations and incident response on a smaller scale and the CSIRT is organized on a higher level, cf. National Institute of Standards and Technology NIST (2020) [19] Security and Privacy Controls for Information Systems and Organizations, Special Publication 800-53, Revision 5, p. 155 s; Association des entreprises électriques suisses AES (2020) [1] Handbuch Grundschutz für “operational Technology” in der Stromversorgung, p. 26; OFEN (2021) [20, p. 133 s].

15 Consid. 34 NIS Directive; AES (2020) [1, p. 24]; ENISA (2020) [8] How to set up a CSIRT and SOC, p. 6; OFEN (2021) [20, p. 133]; Bada M et al. (2014) [2, p. 7 s]; Dsouza (2018) [4, p. 211, 217]. The main reason for outsourcing is, above all for organizations with fewer resources, to get a good level of security at lower cost. A vendor CSIRT is typically a vendor which provides support to commonly used products like operating systems for the public, cf. Dsouza (2018) [4, p. 211].

16 The constituency of a CSIRT is the specific community of clients served by the team, West-Brown et al. (2003) [26, p. 11 f.].

17 The NCSC is the main authority for cybersecurity in Switzerland. It will become a federal office, https://www.ncsc.admin.ch/ncsc/en/home/dokumentation/medienmitteilungen/newslist.msg-id-88878.html (consulted on the 2nd of November 2022).

18 Systems of national importance often include systems of the (federal) administration and of critical infrastructures.

19 ENISA (2015) [7] National/governmental CERTs, ENISA’s recommendations on baseline capabilities, p. 2.

20 Besides GovCERT, SWITCH-CERT has also been recognized as a national CERT but who, in opposition to GovCERT, acts as a private entity, like the other CSIRTs in Switzerland. SWITCH works with specific sectors, SWITCH-CERT, Switzerland’s independent multi-sector CERT, p. 2, https://www.switch.ch/export/sites/default/security/.galleries/files/SWITCH-CERT-Multisektor_en-WEB.pdf (consulted on the 2nd of November 2022).

21 https://www.govcert.admin.ch/ (consulted on the 2nd of November 2022).

22 ENISA (2020) [8] How to set up CSIRT and SOC, p. 6; Dsouza (2018) [4, p. 206 s]. In matter of incident handling, there are other classifications possible, cf. NIST (2012) [18] Computer Security Incident Handling Guide, Recommendations of the National Institute of Standards and Technology, p. 21.

23 OFEN (2021) [20, p. 133]; Dsouza (2018) [4, p. 207].

24 Security quality management services can include risks analysis or general support and advice to an IT department, AES (2020) [1, p. 27 f]; West-Brown et al. (2003) [26, p. 32–34]. “Vulnerability handling involves receiving information and reports about hardware and software vulnerabilities; analyzing the nature, mechanics, and effects of the vulnerabilities; and developing strategies for detecting and repairing the vulnerabilities”, West-Brown et al. (2003) [26, p. 28].

25 CR CO-Chaix (2021) [3, art. 363 N 45].

26 The nFADP has been adopted on the 25 of September 2020 and should enter into force the 1st of September 2023.

27 Art. 19 al. 1 of the Obligation Code.

28 Swiss Federal Counsil (2019) [25] Plan de mise en œuvre de la stratégie nationale de protection de la Suisse contre les cyberrisques (NCS) 2018–2022, p. 32.

29 See Federal Office for National Economic Supply (FONES), Minimum standard for improving ICT resilience.

30 Other subsectors have adopted the ICT minimum standard too, as the public transport, wastewater, etc., cf. https://www.bwl.admin.ch/bwl/en/home/themen/ikt/ikt_minimalstandard/ikt_branchenstandards.html (consulted on 2nd of November 2022).

31 Standards are voluntary unless they have been prescribed by legislation or are the subjects of a contract between several parties, Swiss Federal Counsil (2019) [24] Rapport “Sécurité des produits et gestion des risques de la chaîne d’approvisionnement dans les domaines de la cybersécurité et de la cyberdéfense” en réponse aux postulats Dobler 19.3135 “Acquisitions de l’armée. Avons-nous la maîtrise de la cybersécurité?” et 19.3136 “Infrastructures critiques. Avons-nous la maîtrise des composants matériels et logiciels?” the 18th of March 2019, p. 9.

32 AES (2020) [1, p. 8].

33 AES (2020) [1, p. 26 f.].

34 OFEN (2021) [20, p. 131].

35 NCSC (2022) [17] Modification de la loi fédérale du 18 décembre 2020 sur la sécurité de l’information au sein de la Confédération (loi sur la sécurité de l’information, LSI), Inscription d’une obligation de signaler les cyberattaques contre les infrastructures critiques, Rapport explicatif relatif à l’ouverture de la procédure de consultation, p. 26.

36 See in particular, AES (2020) [1, p. 27 f]; FONES, RC.CO‑3; OFEN (2021) [20, p. 111]; Gilliéron Philippe (2021) [14] L’établissement d’un plan de réponse en cas de faille de sécurité. Other communications could be necessary depending on the nature of the cyber incident, as with human resources in case of phishing.

37 For instance, NIST (2012) [18] Computer security incident handling guide, p. 47; OFEN (2021) [20, p. 160]; West-Brown M et al. (2003) [26, p. 117 f.].

38 Federal Act on the Swiss Financial Market; Finma (2020) [12] Communication FINMA sur la surveillance 05/2020, Obligation de signaler les cyberattaques selon l’art. 29 al. 2 LFINMA, p. 2.

39 Swiss Federal Council, (2017b) [22] Message du 15 septembre 2017 concernant la loi fédérale sur la révision totale de la loi fédérale sur la protection des données et sur la modification d’autres lois fédérales, FF 2017 6680 s. For further information on the report to the FDPIC, see Métille S, Meyer P (2021) [16] Annonce de violations de la sécurité des données: une nouvelle obligation de la nLPD, RSDA 1/2021, p. 23 ff.

40 NCSC (2022) [17] Modification de la loi fédérale du 18 décembre 2020 sur la sécurité de l’information au sein de la Confédération (loi sur la sécurité de l’information, LSI), Inscription d’une obligation de signaler les cyberattaques contre les infrastructures critiques, Rapport explicatif relatif à l’ouverture de la procédure de consultation, p. 22.

41 NCSC (2022) [17] Modification de la loi fédérale du 18 décembre 2020 sur la sécurité de l’information au sein de la Confédération (loi sur la sécurité de l’information, LSI), Inscription d’une obligation de signaler les cyberattaques contre les infrastructures critiques, Rapport explicatif relatif à l’ouverture de la procédure de consultation, p. 21.

42 See for example FIRST, https://www.first.org/about/ (consulted on the 2nd of November 2022).

43 Dsouza Z (2018) [4, p. 214].

44 Addressing resources are defined in art. 3 let. f of the Telecommunications Act (TCA) and may consist of IP addresses, e‑mail addresses or domain names.

45 It should be noted that there are naturally information flows triggered (or not) by the CSIRT but which then take place between different other actors.

46 Swiss Federal Council (2017) [21], Message du 22 février 2017 concernant la loi sur la sécurité de l’information, FF 2017 2873.

47 CR Cst-Dubey J (2021) [5, art. 5 N 35].

48 West-Brown M et al. (2003) [26, p. 62].

49 Swiss Federal Council (2017) [21], Message du 22 février 2017 concernant la loi sur la sécurité de l’information, FF 2017 2801. Critical infrastructure’s operators link voluntarily to this closed client group.

50 GovCERT is habilitated to access the information and IT resources to analyze incidents, even if the operator is bound by legal or contractual confidentiality obligations (art. 74 al. 4 PP-FAIS2). We add that the initial version of the FAIS already stated competencies for GovCERT regarding its support to critical infrastructure’s operators, art. 74 PP-FAIS2.

51 The critical infrastructure’s operators will have to report to the NCSC cyberattacks that meet the conditions of art. 74b ff PP-FAIS2.

52 The provisional nature of this measure is justified by the fact that the latter has no decision-making competence by law, in addition to the fact that the intervention is only based on a suspicion of the competent and recognized service, Federal Office of Communications OFCOM (2017) [11] Rapport explicatif—Révision de l’ordonnance sur les domaines Internet, p. 2. The cases in which the NCSC can act are limited, as for other specialized services (art. 15 al. 3 OID), OFCOM (2017) [11, p. 2].

53 OFCOM (2017) [11, p. 8].

54 And as recalled at art. 12 al. 4 CyRV. GovCERT and the NCSC can be considered as determining the purposes and means of such processing when processing personal data. Their role is not limited to that of a processor, but they really act as a controller, even if the Federal Department of Finance has previously considered the NCSC as a processor, Federal Department of Finances (2020) [10] Erläuterungen zur Verordnung über den Schutz vor Cyberrisiken in der Bundesverwaltung, p. 6.

55 This communication is bidirectional, art. 76 al. 4 PP-FAIS2. In other contexts, GovCERT may also inform the telecommunications service provider concerned when it suspects an abusive use of a Swiss addressing resource, so that the latter can transmit the information to the customers concerned, Swiss Federal Council (2017) [21], Message du 22 février 2017 concernant la loi sur la sécurité de l’information, FF 2017 2872 f.

56 NCSC (2022) [17] Modification de la loi fédérale du 18 décembre 2020 sur la sécurité de l’information au sein de la Confédération (loi sur la sécurité de l’information, LSI), Inscription d’une obligation de signaler les cyberattaques contre les infrastructures critiques, Rapport explicatif relatif à l’ouverture de la procédure de consultation, p. 15.

57 NCSC (2022) [17] Modification de la loi fédérale du 18 décembre 2020 sur la sécurité de l’information au sein de la Confédération (loi sur la sécurité de l’information, LSI), Inscription d’une obligation de signaler les cyberattaques contre les infrastructures critiques, Rapport explicatif relatif à l’ouverture de la procédure de consultation, p. 24 f.

58 NCSC (2022) [17] Modification de la loi fédérale du 18 décembre 2020 sur la sécurité de l’information au sein de la Confédération (loi sur la sécurité de l’information, LSI), Inscription d’une obligation de signaler les cyberattaques contre les infrastructures critiques, Rapport explicatif relatif à l’ouverture de la procédure de consultation, p. 15. GovCERT’s members are not submitted to the obligation to report to prosecuting authorities, to their superior or to the Federal Control of Finances in accordance with art. 22a of the Federal Act on the Confederation’s Personnel (art. 73c al. 2 PP-FAIS2), as this obligation would enter in a confrontation with the confidentiality’s obligation concerning the analysis of an incident, NCSC (2022) [17] Modification de la loi fédérale du 18 décembre 2020 sur la sécurité de l’information au sein de la Confédération (loi sur la sécurité de l’information, LSI), Inscription d’une obligation de signaler les cyberattaques contre les infrastructures critiques, Rapport explicatif relatif à l’ouverture de la procédure de consultation, p. 15.

59 NCSC (2022) [17] Modification de la loi fédérale du 18 décembre 2020 sur la sécurité de l’information au sein de la Confédération (loi sur la sécurité de l’information, LSI), Inscription d’une obligation de signaler les cyberattaques contre les infrastructures critiques, Rapport explicatif relatif à l’ouverture de la procédure de consultation, p. 25. This remote access to the Intelligence Service and the prosecuting authorities can also be given to cantonal security services to necessary information for the protection of cantonal critical infrastructure against cyber risks, art. 76a al. 4 PP-FAIS2.

60 In these contexts, personal data can also be shared when it can be useful to protect critical infrastructure from cyber risks (art. 76 al. 1 PP-FAIS2), NCSC (2022) [17] Modification de la loi fédérale du 18 décembre 2020 sur la sécurité de l’information au sein de la Confédération (loi sur la sécurité de l’information, LSI), Inscription d’une obligation de signaler les cyberattaques contre les infrastructures critiques, Rapport explicatif relatif à l’ouverture de la procédure de consultation, p. 24. Critical infrastructure’s operators can also share this useful personal data with the NCSC and its services, art. 76 al. 2 PP-FAIS2.

61 Swiss Federal Council (2017) [21], Message du 22 février 2017 concernant la loi sur la sécurité de l’information, FF 2017 6657 ff.

62 Swiss Federal Council (2017) [21], Message du 22 février 2017 concernant la loi sur la sécurité de l’information, FF 2017 2874.

63 NCSC (2022) [17] Modification de la loi fédérale du 18 décembre 2020 sur la sécurité de l’information au sein de la Confédération (loi sur la sécurité de l’information, LSI), Inscription d’une obligation de signaler les cyberattaques contre les infrastructures critiques, Rapport explicatif relatif à l’ouverture de la procédure de consultation, p. 14 f.

64 NCSC (2022) [17] Modification de la loi fédérale du 18 décembre 2020 sur la sécurité de l’information au sein de la Confédération (loi sur la sécurité de l’information, LSI), Inscription d’une obligation de signaler les cyberattaques contre les infrastructures critiques, Rapport explicatif relatif à l’ouverture de la procédure de consultation, p. 14.

65 The project doesn’t specify if the competence goes strictly to the NCSC or if GovCERT can also do it, but GovCERT being a part of the NCSC it should not be a problem.

66 National Cyber Security Center NCSC (2022) [17] Modification de la loi fédérale du 18 décembre 2020 sur la sécurité de l’information au sein de la Confédération (loi sur la sécurité de l’information, LSI), Inscription d’une obligation de signaler les cyberattaques contre les infrastructures critiques, Rapport explicatif relatif à l’ouverture de la procédure de consultation, p. 25.

67 Regarding the Traffic Light Protocol, often used for international information sharing, FF 2017 2873; FIRST (2022) [13] Traffic Light Protocol (TLP), FIRST Standards Definitions and Usage Guidance, version 2.0, p. 1 https://www.first.org/tlp/docs/tlp-a4.pdf (consulted on the 2nd of November 2022).
